# The Epigenetic Modifier PRDM5 Functions as a Tumor Suppressor through Modulating WNT/β-Catenin Signaling and Is Frequently Silenced in Multiple Tumors

**DOI:** 10.1371/journal.pone.0027346

**Published:** 2011-11-08

**Authors:** Xing-sheng Shu, Hua Geng, Lili Li, Jianming Ying, Chunhong Ma, Yajun Wang, Fan Fong Poon, Xian Wang, Ying Ying, Winnie Yeo, Gopesh Srivastava, Sai Wah Tsao, Jun Yu, Joseph J. Y. Sung, Shi Huang, Anthony T. C. Chan, Qian Tao

**Affiliations:** 1 Cancer Epigenetics Laboratory, Department of Clinical Oncology, Sir YK Pao Center for Cancer and Li Ka Shing Institute of Health Sciences, The Chinese University of Hong Kong, and CUHK Shenzhen Research Institute, Hong Kong, China; 2 Department of Pathology, Cancer Hospital, Peking Union Medical College & Chinese Academy of Medical Sciences, Beijing, China; 3 Shandong University School of Medicine, Shandong, China; 4 Department of Pathology, University of Hong Kong, Hong Kong, China; 5 Department of Anatomy, University of Hong Kong, Hong Kong, China; 6 Institute of Digestive Disease and Department of Medicine, The Chinese University of Hong Kong, Hong Kong, China; 7 State Key Laboratory of Medical Genetics, Xiangya Medical School, Central South University, Changsha, China; Northwestern University Feinberg School of Medicine, United States of America

## Abstract

**Background:**

PRDM (PRDI-BF1 and RIZ domain containing) proteins are zinc finger proteins involved in multiple cellular regulations by acting as epigenetic modifiers. We studied a recently identified PRDM member PRDM5 for its epigenetic abnormality and tumor suppressive functions in multiple tumorigeneses.

**Methodology/Principal Findings:**

Semi-quantitative RT-PCR showed that *PRDM5* was broadly expressed in human normal tissues, but frequently silenced or downregulated in multiple carcinoma cell lines due to promoter CpG methylation, including 80% (4/5) nasopharyngeal, 44% (8/18) esophageal, 76% (13/17) gastric, 50% (2/4) cervical, and 25% (3/12) hepatocellular carcinoma cell lines, but not in any immortalized normal epithelial cell lines. *PRDM5* expression could be restored by 5-aza-2′-deoxycytidine demethylation treatment in silenced cell lines. *PRDM5* methylation was frequently detected by methylation-specific PCR (MSP) in multiple primary tumors, including 93% (43/46) nasopharyngeal, 58% (25/43) esophageal, 88% (37/42) gastric and 63% (29/46) hepatocellular tumors. *PRDM5* was further found a stress-responsive gene, but its response was impaired when the promoter was methylated. Ectopic *PRDM5* expression significantly inhibited tumor cell clonogenicity, accompanied by the inhibition of TCF/β-catenin-dependent transcription and downregulation of *CDK4*, *TWIST1* and *MDM2* oncogenes, while knocking down of *PRDM5* expression lead to increased cell proliferation. ChIP assay showed that PRDM5 bound to its target gene promoters and suppressed their transcription. An inverse correlation between the expression of *PRDM5* and activated β-catenin was also observed in cell lines.

**Conclusions/Significance:**

PRDM5 functions as a tumor suppressor at least partially through antagonizing aberrant WNT/β-catenin signaling and oncogene expression. Frequent epigenetic silencing of *PRDM5* is involved in multiple tumorigeneses, which could serve as a tumor biomarker.

## Introduction

Tumor-specific epigenetic silencing of tumor suppressor genes (TSGs) through promoter CpG methylation and histone modification is frequently involved in multiple carcinogenesis [1]. CpG methylation can also be used as an epigenetic biomarker for novel TSG identification and tumor diagnosis. A series of TSGs such as *p16*, *RASSF1A*, *PCDH10* and *RASAL*, have been identified with epigenetic inactivation in multiple cancers, through promoter CpG methylation [2]–[4].

PRDM (PRDI-BF1 and RIZ domain containing) proteins belong to the zinc finger protein family, harboring an evolutionarily conserved N-terminal PR domain followed by 16 zinc finger repeats [5]. The PR domain, a characteristic C2-H2 zinc finger motif, shares high homology with SET (Suvar3–9, Enhancer-of-zeste, Trithorax) domain that is involved in chromatin-mediated transcriptional regulation [6]. Thus far, seventeen human *PRDM* members have been identified, with several possessing growth inhibitory functions in tumor cells. PRDM1 (PRDI-BF1 or BLIMP1) functions as a transcriptional repressor of *c-MYC* and induces cell differentiation and apoptosis [7], [8]. PRDM2 (RIZ1) is the first identified member of methyltransferase family with tumor suppressive function mediated by its PR domain [9], [10]. The long isoform of *PRDM3* (*MDS1-EVI1*) is commonly mutated in myeloid leukemia [11]. *PRDM4* (*PFM1*) is located at a tumor suppressor locus 12q23-q24.1 commonly deleted in ovarian, gastric and pancreatic cancers [12].

*PRDM5* (also known as *PFM2*) is a recently identified PRDM member, mapped to a commonly deleted region 4q25-26 [13]. PRDM5 was first identified from an EST database based on its conserved PR domain at the N-terminus followed by 16 zinc finger motifs [14]. It acts as a candidate TSG in some tumors [14], [15]. Recent reports showed *PRDM5* silencing by promoter methylation in multiple cancers including breast, ovarian, colorectal, gastric, and hepatocellular tumors. Ectopic expression of PRDM5 leads to G2/M arrest and apoptosis of tumor cells [14], although its molecular mechanism is still unclear. PRDM5 is a nuclear protein involved in the epigenetic regulation of various genes, through interaction with histone methyltransferase G9A and histone deacetylase 1 (HDAC1) [16].

Here, we studied the epigenetic abnormality and tumor suppressive functions of PRDM5 in multiple common tumors including nasopharyngeal, esophageal, gastric, hepatocellular and cervical cancers.

## Results

### Frequent silencing of *PRDM5* in multiple tumor cell lines due to promoter methylation

**Figure 1:**
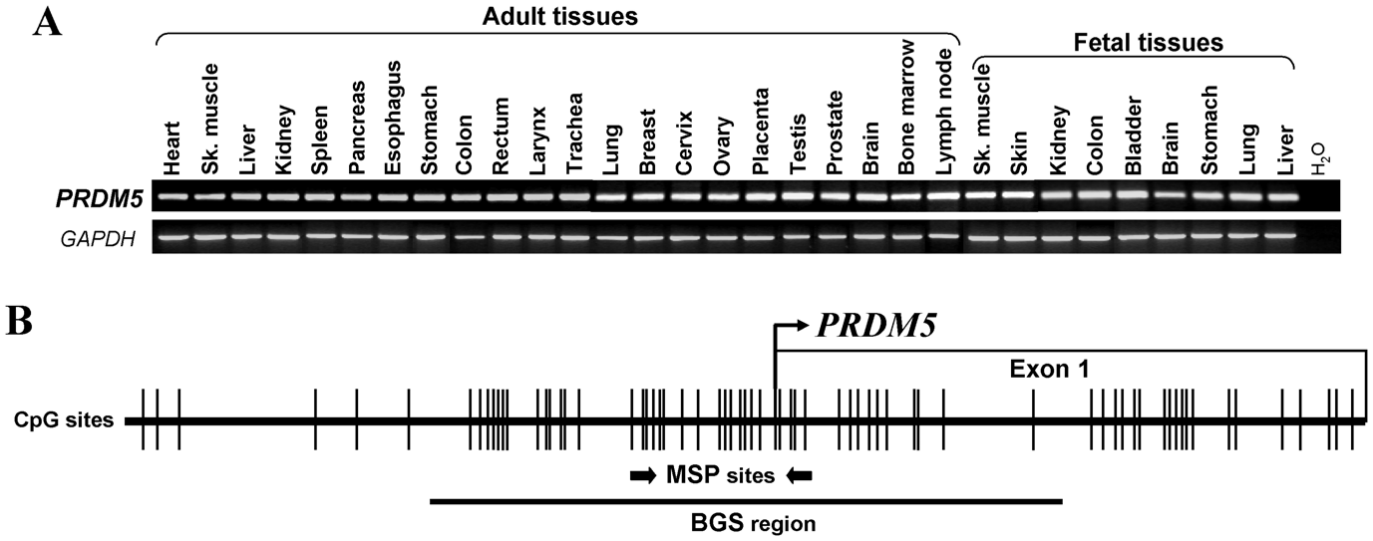
*PRDM5* expression profile in normal tissues and the CpG island in its promoter. (A) *PRDM5* is broadly expressed in human normal adult and fetal tissues. Sk, skeleton. (B) *PRDM5* promoter contains a typical CpG island. Each CpG site is shown as a vertical bar, MSP and BGS primers are indicated. Transcription start site is showed by a curved arrow.

**Figure 2:**
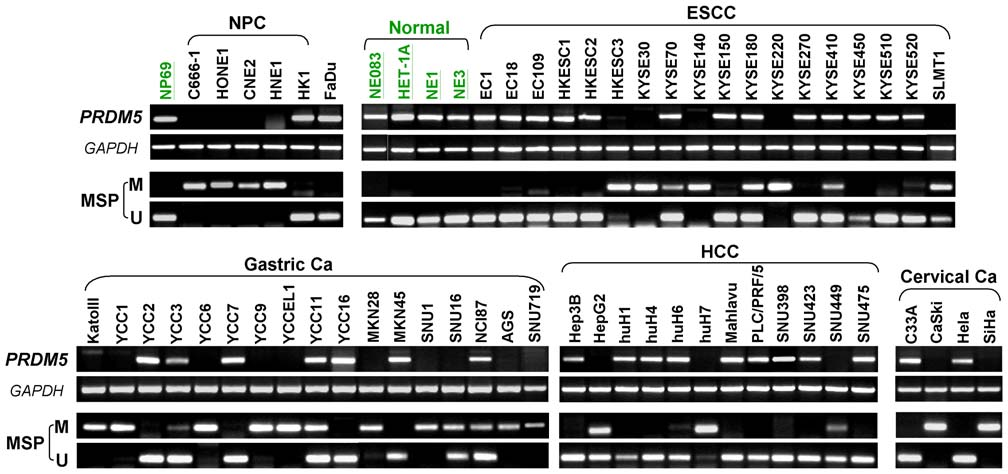
Silencing of *PRDM5* by promoter methylation. *PRDM5* is frequently silenced and methylated in multiple carcinoma cell lines, while expressed and unmethylated in all immortalized normal epithelial cell lines examined (underlined) with *GAPDH* as a control. Ca, carcinoma; NPC, nasopharyngeal carcinoma; ESCC, esophageal squamous cell carcinoma; HCC, hepatocellular carcinoma; M, methylated; U, unmethylated.

We first examined *PRDM5* expression in a panel of human normal adult and fetal tissues by semi-quantitative RT-PCR. Results showed that *PRDM5* was broadly expressed in all normal tissues (Figure 1A). We then checked *PRDM5* expression in multiple carcinoma cell lines. Results revealed that *PRDM5* was frequently downregulated or silenced in cell lines of nasopharyngeal, esophageal, gastric, hepatocellular and cervical carcinomas, but readily detected in immortalized normal epithelial cell lines (Figure 2).

There is a typical CpG island (CGI) spanning the transcription start site of *PRDM5* (Figure 1B), as predicted by CGI searcher (http://cpgislands.usc.edu/). We thus analyzed its promoter CpG methylation in tumor cell lines. Methylation-specific PCR (MSP) results showed that *PRDM5* was frequently methylated in silenced cell lines, including 80% (4/5) nasopharyngeal, 44% (8/18) esophageal, 76% (13/17) gastric, 25% (3/12) hepatocellular, and 50% (2/4) cervical carcinoma cell lines (Figure 2, Table 1), with the exception of only infrequent *PRDM5* methylation detected in lung, colon, ovarian and bladder cancer cell lines (Figure S1). In contrast, no methylation was observed in immortalized normal epithelial cell lines (Figure 2, Table 1).

**Table 1:** Summary of *PRDM5* methylation in cell lines and primary tumors.

Samples	Promoter methylation (%)
**Carcinoma cell lines**	
Nasopharyngeal	80% (4/5)
Esophageal	44% (8/18)
Gastric	76% (13/17)
Cervical	50% (2/4)
Hepatocellular	25% (3/12)
Colorectal	27% (3/11)
Lung	29% (2/7)
Ovarian	0/2
Bladder	2/3
**Primary tumors**	
Nasopharyngeal Ca	93% (43/46)
Esophageal Ca	58% (25/43)
Gastric Ca	88% (37/42)
Hepatocellular Ca	63% (29/46)

**Figure 3:**
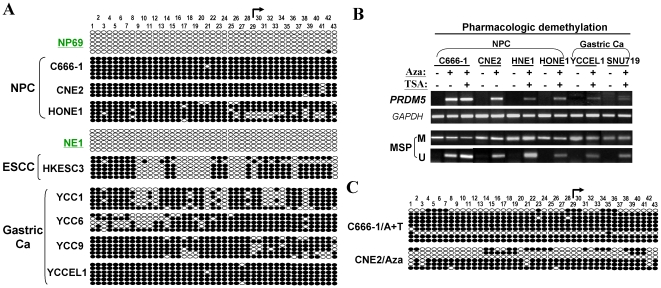
Pharmacologic demethylation restores *PRDM5* expression. (A) High-resolution methylation analysis of *PRDM5* promoter by BGS. Each row of circles represents an individual promoter allele. Filled circle, methylated CpG site; open circle, unmethylated CpG site. (B) Pharmacologic demethylation by Aza alone or combined with TSA restores *PRDM5* expression in methylated and silenced carcinoma cell lines. (C) Detailed BGS analysis of *PRDM5* promoter demethylation in cell lines treated with Aza alone or combined with TSA.

To confirm the MSP results, we further examined *PRDM5* promoter methylation by high-resolution bisulfite genomic sequencing (BGS) analysis of 43 individual CpG sites within its CGI. The BGS results were consistent with those of MSP, with all promoter alleles intensively methylated in silenced cell lines but rare methylated CpG sites detected in expressing normal cell lines (Figure 3A). Taken together, these results revealed a strong correlation between *PRDM5* promoter CpG methylation and its transcriptional silencing in tumor cell lines.

### Pharmacologic demethylation restores *PRDM5* expression

To determine whether promoter methylation directly contributes to *PRDM5* silencing, several silenced tumor cell lines were treated with DNA methyltransferase inhibitor 5-aza-2′-deoxycytidine (Aza) alone or combined with histone deacetylase inhibitor trichostatin A (TSA). This pharmacologic demethylation restored *PRDM5* expression in all silenced cell lines, accompanied by the increase of demethylated promoter alleles (Figure 3B and 3C), indicating that *PRDM5* silencing in tumor cells was directly mediated by promoter methylation.

### *PRDM5* is a stress responsive gene but its response is disrupted by promoter methylation

**Figure 4:**
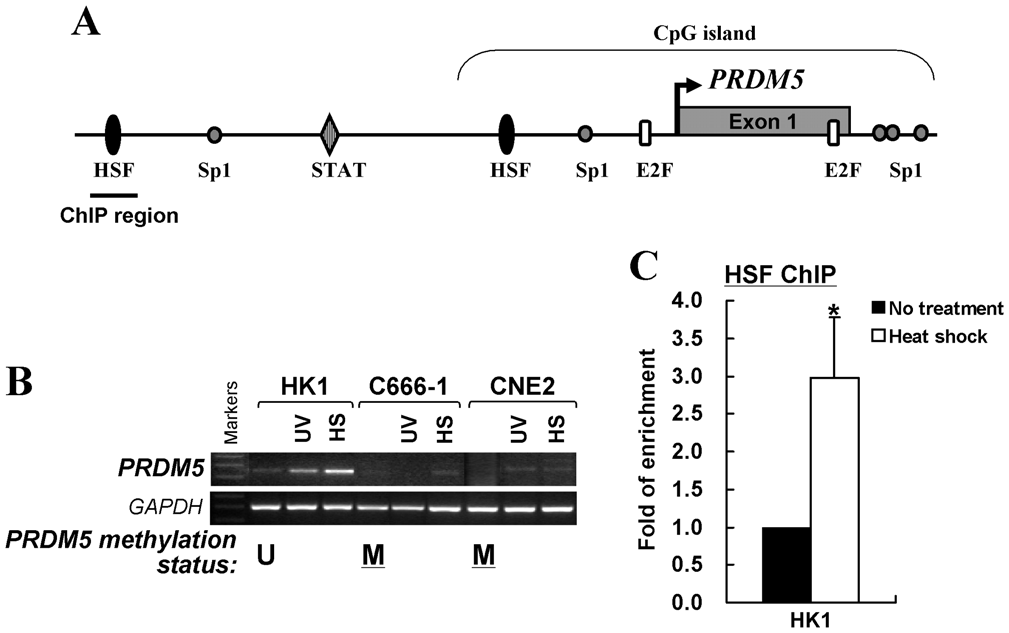
*PRDM5* is stress responsive. (A) Transcription factor binding sites in the *PRDM5* promoter. Promoter region used for ChIP assay was indicated. (B) Induction of *PRDM5* in response to stresses was disrupted in methylated cell lines, but not in unmethylated ones. Promoter methylation status was shown at the bottom. M, methylated; U, unmethylated. (C) ChIP assay showed enrichment of HSF1 binding to *PRDM5* promoter after heat shock treatment in HK1 cells. *p<0.05.

Analysis of regulatory elements of the *PRDM5* promoter by TFSearch (www.cbrc.jp/research/db/TFSEARCH) revealed two HSF (heat shock factor) and five Sp1 binding sites (Figure 4A), suggesting that *PRDM5* might be a stress-responsive gene. We then examined its response to environmental stress stimuli. We found that *PRDM5* expression was dramatically induced in HK1 cells with unmethylated promoter alleles, after exposure to heat shock or UV irradiation. However, this stress response was significantly reduced or totally abolished in cell lines with methylated promoter (C666-1 and CNE2) (Figure 4B). Moreover, ChIP assay showed significant enrichment of HSF1 binding to *PRDM5* promoter after heat shock treatment of HK1 cells (Figure 4C), suggesting that *PRDM5* is indeed stress-responsive but this response is disrupted when the promoter is methylated.

### *PRDM5* is frequently methylated in primary tumors

**Figure 5:**
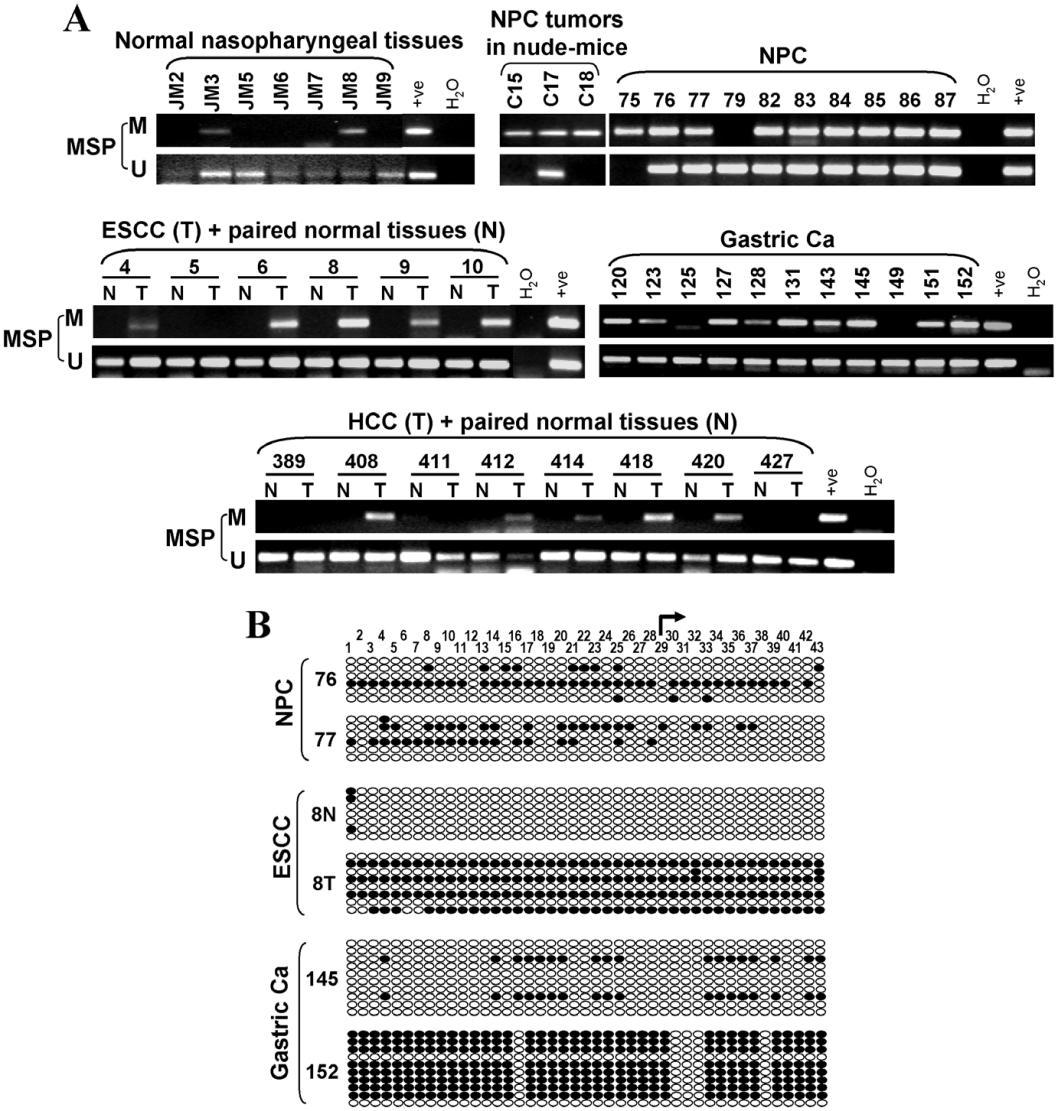
Frequent methylation of *PRDM5* in primary tumors. (A) Representative MSP analysis in primary tumors and normal tissues. M, methylated; U, unmethylated. N, paired adjacent normal tissue; T, tumor. (B) Detailed analysis of *PRDM5* methylation in primary tumors by BGS.

**Figure 6:**
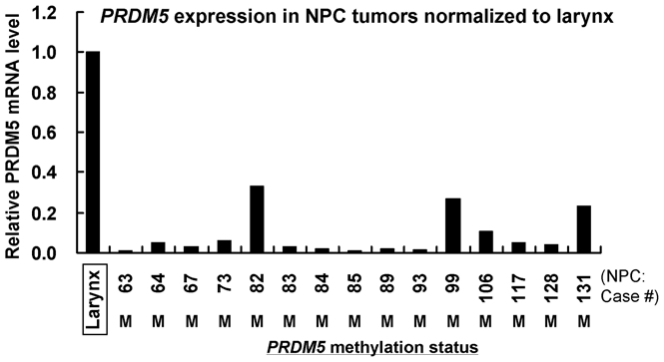
*PRDM5* expression in primary NPC tumors determined by qRT-PCR. *PRDM5* expression level in each sample was normalized to internal control *GAPDH*. *PRDM5* expression level in each tumor sample relative to normal larynx is shown, with *PRDM5* methylation status shown at the bottom. M, methylated.

We next examined *PRDM5* methylation in primary tumors. MSP detected *PRDM5* methylation in 93% (43/46) nasopharyngeal, 58% (25/43) esophageal, 88% (37/42) gastric and 63% (29/46) hepatocellular carcinomas. In contrast, only weak methylation was infrequently detected in normal tissues (2/7 nasopharyngeal and 3/7 esophageal tissues), while no methylation was detected in any of the 9 paired normal tissues of HCC (Figure 5A, Table 1). BGS analysis confirmed the dense methylation of *PRDM5* in primary tumors but not normal tissues (Figure 5B). Moreover, quantitative RT-PCR analysis revealed that *PRDM5* was frequently downregulated in methylated primary NPC tumors when compared to normal larynx (Figure 6).

### PRDM5 inhibits tumor cell growth and proliferation

**Figure 7:**
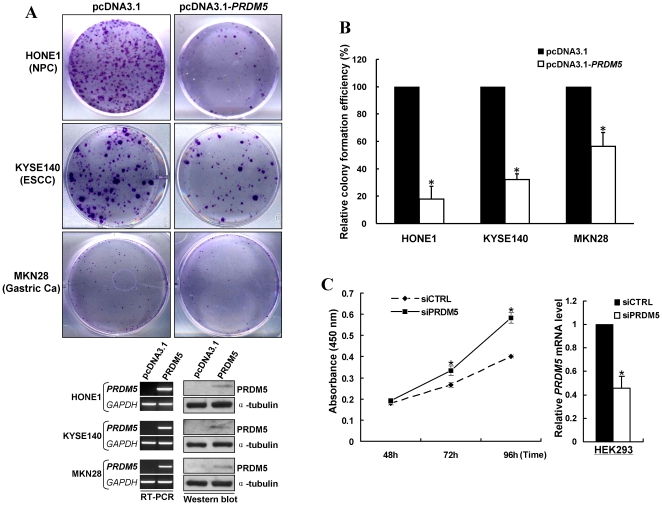
PRDM5 inhibits tumor cell growth and proliferation. (A) Representative colony formation assay of HONE1, KYSE140 and MKN28 cell lines (upper panel); confirmation of ectopic expression of transfected *PRDM5* in these cell lines by RT-PCR and Western blot (bottom panel). (B) Quantitative analysis of colony formation assay. Data were presented as means±SD of three independent experiments. (C) Knock-down of endogenous *PRDM5* using siRNA in HEK293 cells significantly increased cell proliferation rate as determined by CCK8 assay (left); *PRDM5* mRNA levels of HEK293 cells were evaluated by qRT-PCR (right). siCTRL, control siRNA; siPRDM5, PRDM5 siRNA.

Frequent silencing of *PRDM5* in multiple cancer cell lines and primary tumors indicated that *PRDM5* likely functions as a tumor suppressor. Thus, we examined the effects of ectopic *PRDM5* expression on tumor cell clonogenicity. A mammalian expression vector encoding full-length *PRDM5* was transfected into nasopharyngeal, esophageal and gastric cancer cell lines with completely methylated and silenced *PRDM5* (HONE1, KYSE140 and MKN28). Ectopic *PRDM5* expression dramatically reduced the colony formation efficiencies of all cell lines in monolayer culture, as compared to vector controls (down to 18%, 32% and 56%, respectively, Figure 7A upper panel and 7B). Expression of *PRDM5* in these cell lines was confirmed by RT-PCR and Western blot (Figure 7A bottom panel). Consistently, knock-down of endogenous *PRDM5* using siRNA in HEK293 cells significantly increased the cell proliferation rate (Figure 7C), suggesting that *PRDM5* does function as a tumor suppressor.

### PRDM5 modulates WNT/β-catenin signaling and represses oncogene expression

**Figure 8:**
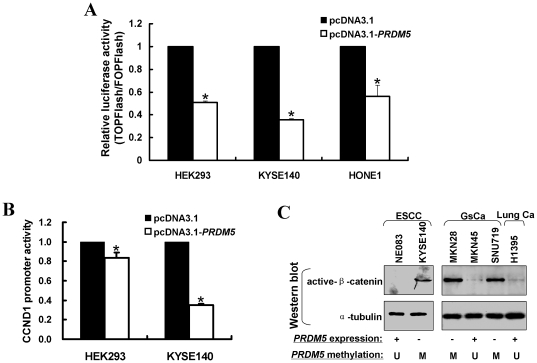
PRDM5 antagonizes WNT/β-catenin signaling. (A) Effects of ectopic *PRDM5* expression on WNT/β-catenin signaling were assessed by TOPFlash luciferase reporter assay, with FOPFlash reporter as a control. (B) Ectopic expression of *PRDM5* repressed *CCND1* promoter (+ ∼2 kb upstream) activity by as determined dual-luciferase assay. (C) Correlation of active β-catenin expression and *PRDM5* methylation/silencing in carcinoma cell lines. Active β-catenin protein level was determined by Western blot. *PRDM5* expression was evaluated by RT-PCR. +, expressed; -, silenced. *PRDM5* promoter methylation was examined by MSP. M, methylated; U, unmethylated.

As a zinc finger transcription factor and epigenetic modifier, PRDM5 should exert its tumor suppressive functions through modulating cell signaling and gene transcription. We thus analyzed the effect of PRDM5 expression on cell signaling using the TCF/β-catenin- (TOPFlash) and Ras/MAPK-ERK- signaling (SRE) luciferase reporter constructs. Although PRDM5 expression did not affect SRE luciferase reporter activity (data not shown), it did significantly inhibit TOPFlash reporter activity, an indicator of TCF/LEF-dependent transcription, with FOPFlash reporter construct containing mutant TCF/LEF binding sites as a control (Figure 8A). Moreover, the promoter reporter activity of *CCND1*, a WNT/β-catenin downstream target gene, was markedly decreased when PRDM5 was expressed (Figure 8B). We also examined whether *PRDM5* methylation/silencing is correlated with enhanced WNT/β-catenin signaling in tumor cell lines, and found that the active form of β-catenin was accumulated in cell lines with methylated and silenced *PRDM5*, with significant lower level of active β-catenin detected in *PRDM5*-expressing cell lines (Figure 8C).

**Figure 9:**
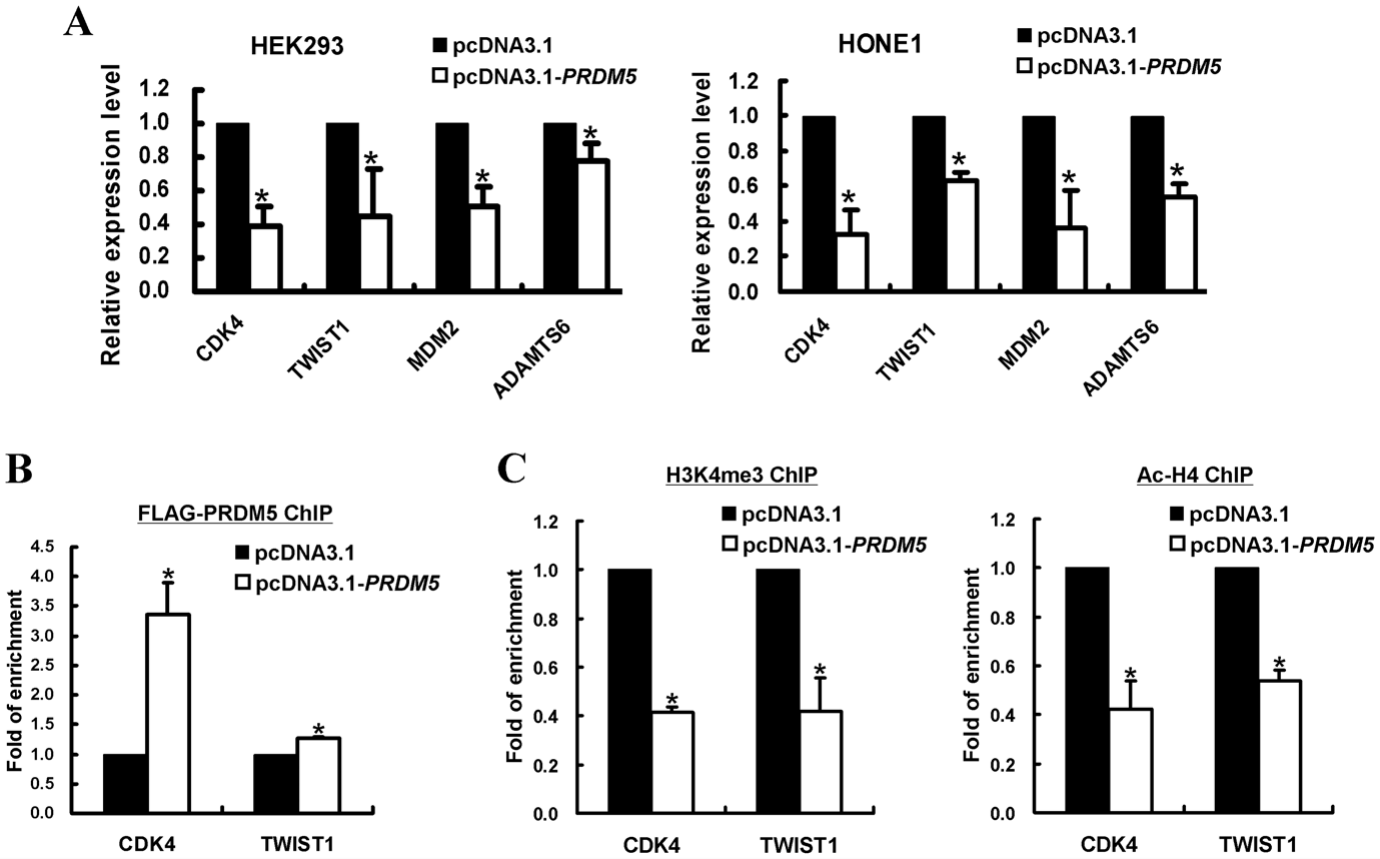
PRDM5 suppresses multiple oncogenes expression. (A) Suppression of oncogene expression by PRDM5 in HEK293 and HONE1 cells, evaluated by real-time PCR. (B) ChIP assay in HEK293 cells showed direct association of PRDM5 with *CDK4* and *TWIST1* promoters. (C) Downregulation of *CDK4* and *TWIST1* by PRDM5 expression is accompanied by decreased H3K4me3 and acetyl-histone H4 levels at their promoters, determined by ChIP assay in HEK293 cells.

**Figure 10:**
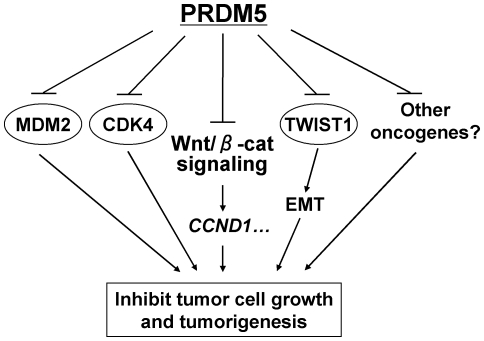
Proposed model of the tumor suppressive functions of PRDM5. PRDM5 antagonizes WNT/β-catenin signaling and suppresses oncogene expression (CDK4, TWIST1, MDM2, etc), which leads to tumor suppression.

We further examined the expression changes of multiple oncogenes and tumor suppressor genes after ectopic PRDM5 expression in both normal and tumor cell lines (HEK293 and HONE1). Real-time PCR showed that several oncogenes were dramatically repressed by PRDM5, such as *CDK4*, *TWIST1*, and *MDM2* (Figure 9A, Table S1). We further performed ChIP assay, and found direct binding of PRDM5 to the promoters of *CDK4* and *TWIST1* (Figure 9B). Moreover, PRDM5 expression resulted in significant decreased levels of H3K4me3 and acetyl-histone H4 in *CDK4* and *TWIST1* promoters (Figure 9C), both as active transcription marks. Thus, PRDM5 does exert its tumor suppressive functions through modulating WNT/ β-catenin signaling and the expression of multiple oncogenes as an epigenetic modifier (Figure 10).

## Discussion

Identification of TSGs silenced by CpG methylation elucidates the molecular mechanisms of carcinogenesis and develops epigenetic biomarkers for cancer detection and prognosis prediction. In the present study, we showed that *PRDM5,* an epigenetic modifier gene, was frequently inactivated by promoter methylation in multiple common cancers. Ectopic expression of *PRDM5* inhibited tumor cell clonogenicity, at least partially through antagonizing WNT/β-catenin signaling and oncogene expression such as *CDK4*, *TWIST1*, and *MDM2*.

Epigenetic silencing of TSGs is critically involved in the onset and progression of multiple cancers, even preceding genetic changes during tumorigenesis [17]. CpG methylation of TSG promoters is a fundamental epigenetic mechanism leading to their inactivation in tumors [1]. Previous studies showed that *PRDM5* silencing was mediated by either DNA methylation or trimethylation of H3K27 [15], but with limited cell lines and tumors studied. Here, we systematically studied *PRDM5* silencing and methylation in a large collection of cell lines and primary tumors. Our results showed that aberrant methylation of *PRDM5* was associated with its inactivation in multiple tumor cell lines and primary tumors, with the silencing/methylation of *PRDM5* in cell lines of gastric cancer (MKN45) and HCC (huH1, Hep3B and HepG2) consistent with previous studies [14], [15]. Our results demonstrate that tumor-specific promoter methylation represents the predominant mechanism of *PRDM5* inactivation, although other mechanisms like gene micro-deletion or mutations might also exist.

We also found that *PRDM5* is a stress-responsive gene, but its response is impaired when the promoter is methylated. When inspecting the *PRDM5* promoter, we found two predicted HSF binding sites, with the one at −390 to −380 (**CG** GAAATTTCC) containing a CpG site. It is likely that methylation of this CpG site would affect the binding of HSF, leading to the disruption of its stress response. Thus, epigenetic silencing of *PRDM5* could abolish cellular protective response to environmental stresses, contributing to tumorigenesis.

PRDM5 is a zinc finger protein. As a sequence-specific DNA-binding transcription factor, PRDM5 functions as a repressor targeting multiple protein-coding and miRNA genes involved in cell cycle, signal transduction and protein modification [16]. Although with a PR/SET domain in the N-terminus, PRDM5 itself does not possess histone methyltransferase (HMTase) activity. Instead, PRDM5 acts as an epigenetic modifier by recruiting histone-modifying enzymes like HMTase G9A and HDAC1 to target genes [16]. Our study indicates that if the epigenetic modifier PRDM5 is inactivated in tumor cells, its repression to target oncogenes would be released and thereby contributing to tumor initiation and progression.

Previous report showed that ectopic PRDM5 expression caused cell cycle G2/M arrest and induced apoptosis in tumor cells [14], while the molecular mechanisms remain unknown. Recently, the zebrafish PRDM5 was found to regulate Wnt/β-catenin signaling at the early stages of zebrafish development [18]. Our results are in agreement with this study. Aberrant activation of WNT/β-catenin signaling is frequently involved in cancers, accompanied with elevated levels of active β-catenin [19]. In addition to genetic defects, epigenetic silencing of WNT/β-catenin antagonists also leads to aberrant WNT/β-catenin signaling in tumors [19]. Frequent methylation-mediated silencing of extracellular (*SFRPs* and *DKKs*) [20]–[22] and cytosolic (*APC*, *AXIN2* and *DACT3*) [23]–[25] WNT antagonists, nuclear proteins (*SOX7* and *SOX17*) [26], [27], and non-transforming WNT families (*WNT5A*, *WNT7A* and *WNT9A*) occurs in cancers [28]–[30], indicating that the epigenetic inactivation of WNT-signaling negative regulators plays a critical role in tumor pathogenesis. Our finding that ectopic expression of PRDM5 leads to the inhibition of WNT/β-catenin signaling in tumor cells, probably through upregulating *DKK1*, *DKK2*, and *WNT5A* [18], suggests a novel mechanistic link between PRDM5 and WNT signaling in human tumorigenesis. PRDM5 also significantly inhibited the promoter activity of *CCND1*, a direct downstream target oncogene of WNT/β-catenin signaling which acts as a mitogenic signal sensor [31], [32]. CDK4 is one of the binding partners of CCND1 and also transcriptionally downregulated by PRDM5. *PRDM5* silencing is also linked to the accumulation of active β-catenin in tumor cells, which leads to constitutive activation of WNT/β-catenin signaling.

In summary, we found that *PRDM5* was frequently silenced by promoter methylation in multiple tumors. PRDM5 suppresses tumor cell growth through antagonizing WNT/β-catenin signaling and suppressing multiple oncogenes expression. The high incidence of *PRDM5* methylation in some carcinomas indicates that it is a potential epigenetic biomarker for these tumors.

## Materials and Methods

### Cell lines and tumor samples

A series of tumor cell lines were used, including nasopharyngeal, esophageal, gastric, hepatocellular, lung, colorectal, and cervical cell lines [33], [34]. Immortalized epithelial cell lines NP69, NE083, Het-1A, NE1 and NE3 were used as normal controls. Carcinoma cell lines were maintained in RPMI 1640 (Invitrogen) supplemented with 10% fetal bovine serum. Cell lines were purchased from the American Type Culture Collection (ATCC, Manassas, VA) or obtained from collaborators. Human normal adult and fetal tissue RNA samples were purchased commercially (Stratagene, La Jolla, CA or Millipore Chemicon, Billerica, MA). Samples of normal nasopharyngeal and esophageal epithelial tissues from healthy individuals were described previously [33]. Nude mice-passaged undifferentiated NPC tumors from North Africans, C15, C17 and C18 were also used [34]. DNA samples of NPC, gastric tumors, esophageal squamous cell carcinomas, hepatocellular carcinomas (T) and their corresponding surgical marginal normal tissues (N), were described previously [34].

### Pharmacologic demethylation and stress treatments

Cell lines were treated with DNA methyltransferase inhibitor 5-aza-2′-deoxycytidine (Aza) (Sigma, St. Louis, MO) and histone deacetylase inhibitor Trichostatin A (TSA) as reported previously [3]. Cells were subjected to heat shock treatment with incubation at 42^o^C for 1 hour followed by recovery at 37°C for 2 hours. For UV treatment, medium was removed and the flask was turned upside down to face the light source in a UV cross-linker (Amersham Biosciences, Piscataway, NJ). Cells were irradiated for a dose of 70 J/m^2^. After irradiation, fresh medium was added, and cells were recovered at 37°C for 1 hour and then harvested [33].

### Plasmid construction

The full length open reading frame (ORF) of *PRDM5* [14] was subcloned into the *NotI* and *KpnI* site of the pcDNA3.1 mammalian expression vector to generate pcDNA3.1-*PRDM5* with sequence and orientation confirmed.

### Semi-quantitative RT-PCR and real-time PCR

Total RNA was extracted from cell lines using TRI reagent. Reverse transcription (RT) using random hexamer, and RT-PCR using Go-Taq (Promega, Madison, WI) were performed as previously, with *GAPDH* as a control [34]. Primers used were PRDM5F: 5′-CAGGTTCTCCCTGAAGTCCT and PRDM5R: 5′-TGAGATGGTGCCTCATGAAC. RT-PCR was performed for 32 cycles for *PRDM5*, while 23 cycles for *GAPDH*.

Real-time PCR was performed using SYBR Green master mixture and an HT7900 system according to the manufactures' instruction (Applied Biosystems, Foster City, CA). To screen for PRDM5 modulated target genes, total RNA extracted from *PRDM5* or vector-transfected cells was treated with DNase I, and reverse transcripted as described previously [34]. Real-time PCR was performed for 34 selected candidate target genes. For each gene, the expression level in *PRDM5*-transfected cells was normalized to control. Primer sequences were listed in Table S2.

### Methylation-specific PCR (MSP) and bisulfite genomic sequencing (BGS)

Bisulfite modification of DNA, MSP and BGS was performed as previously described [4]. The bisulfite-treated DNA was amplified with methylation-specific primer set, PRDM5m1: 5′-TTGTTTCGGGTTTCGCGTTC, PRDM5m2: 5′-ATTCCTACTACGAAAACGCG; or unmethylation-specific primer set, PRDM5u11: 5′-TAGTTTTGTTTTGGGTTTTGT, PRDM5u2: 5′-CCATTCCTACTACAAAAACACA. All MSP primers were tested for not amplifying any unbisulfited genomic DNA to ensure the specificity of MSP. For BGS, bisulfite-treated DNA was amplified using BGS primer set, PRDM5BGS3: 5′-GTTTGAAAATTTAGAGTTGGAT and PRDM5BGS4: 5′-ACCAAAATAAAAAAAAAAAACC. The BGS PCR products were TA-cloned into pCR4-TOPO vector with 4–10 colonies randomly chosen for sequencing.

### Chromatin immunoprecipitation (ChIP)

ChIP assay was performed using a commercial kit (17–295, Upstate) according to manufacturer's protocol. 1 µg antibody was used for each ChIP assay, while no-antibody immunoprecipitation was used as negative control. Input DNA and immunoprecipitated DNA were purified with QIAamp DNA mini kit (51306, Qiagen). The enrichment of each target sequence was determined by real-time PCR. Immunoprecipitated DNA enrichment was normalized to its input. Antibodies used were HSF1 (4356, Cell Signaling), FLAG M2 (F3165, Sigma), Histone H3K4me3 (ab8580, Abcam), and acetyl-Histone H4 (06–866, Upstate). Primers used were: PRDM5ChIPF: 5′-TGGAAAACCTACTTAGTCCAG and PRDM5ChIPR: 5′-GAGGCTGATCTGAGATGTTC; CDK4ChIPF: 5′-TACACACTGGAAGCAAGCAC and CDK4ChIPR: 5′-GGTCTTTCAGCCTGTCTGC; TWIST1ChIPF: 5′-GGAGGACGAATTGTTAGACC and TWIST1ChIPR: 5′-GCAGTGTCATTGGCCTGAC.

### Colony formation assay

Cells were seeded at 1×10^5^ cells/ml and cultured overnight in 12-well plates. Cells were then transfected with pcDNA3.1-*PRDM5* or empty vector using FuGene6 (Roche). Forty-eight hours post-transfection, cells were collected and seeded in 6-well plates at an appropriate density with G418 selection (400 µg/ml). After 2-3 weeks, cells were stained with Gentian Violet with survived colonies (≥50 cells/colony) counted.

### siRNA transfection and cell proliferation assay

*PRDM5* siRNA and siRNA negative control were obtained from Invitrogen. siRNAs were transfected using Lipofectamine 2000 (Invitrogen) according to manufacturer's protocol. Twenty-four hours after transfection, cells were collected and seeded in 96-well plates (10^3^ cells per well). Cell proliferation rates were determined at indicated time points using Cell Counting Kit-8 (Dojindo, Rockville, MD). Triplicates for each sample were performed.

### Dual-luciferase reporter assay

Luciferase reporter construct of *CCND1* promoter (+ ∼2 kb upstream of transcription start site), or TOPFlash reporter containing 4xTCF-binding sites (kind gift from Dr. Christof Niehrs, German Cancer Research Center (DKFZ)), or FOPFlash reporter containing mutant TCF/LEF binding sites (kind gift from Dr. Jin Dong-Yan, University of Hong Kong) was co-transfected with either pcDNA3.1-*PRDM5* or empty vector, together with an internal control Renilla luciferase reporter pRL-CMV vector using FuGene6 [35]. Forty-eight hours after transfection, cells were harvested and analyzed by the dual-luciferase assay kit (Promega, Madison, WI). Each experiment was repeated for three times. Statistical analysis was carried out by Student's *t*-test, *p*<0.05 was considered as statistically significant difference.

### Western blot

Cell pellets were incubated in lysis buffer (50 mmol/L Tris-HCl, pH 8.0; 150 mmol/L NaCl; 0.5% NP40) for 30 minutes on ice, followed by centrifugation at 14,000 rpm for 15 minutes at 4°C. Cell lysates were resolved using SDS-PAGE gels and transferred onto nitrocellulose membranes. Membranes were then incubated with primary antibodies for 1 hour at room temperature or overnight at 4°C, followed by incubation with a secondary antibody at room temperature for 1 hour. Immunoreactive bands were detected using Western blot Luminol reagent (GE Healthcare Bio-Sciences) according to the manufacturer's protocol. Antibody used were FLAG M2 (F3165, Sigma), active β-catenin (05-665, Upstate) and α-Tubulin (DM1A, Lab Vision).

## Supporting Information

Figure S1 *PRDM5* downregulation and methylation is rarely detected in cell lines of lung, colorectal, ovarian and bladder cancer. Ca, carcinoma; M, methylated; U, unmethylated.
(TIF)

Table S1 Real-time PCR of PRDM5 target genes. Modulation of multiple genes by PRDM5 expression in HEK293 and HONE1 cells was evaluated by real-time PCR. Data were obtained from three independent experiments and presented as relative average ratio. The expression of target genes in PRDM5-transfected cells were normalized to those in vector controls, GAPDH was used as internal control. p<0.05 was considered as statistical significance by Student's t-test.
(DOC)

Table S2 Primers for target gene screening by real-time PCR analysis.
(DOC)
