# Supplementary figures and images for: The Epigenetic Modifier PRDM5 Functions as a Tumor Suppressor through Modulating WNT/β-Catenin Signaling and Is Frequently Silenced in Multiple Tumors
Source: PLoS One. 2011 Nov 8;6(11):e27346. doi: 10.1371/journal.pone.0027346 (PMC3210799; doi:10.1371/journal.pone.0027346)

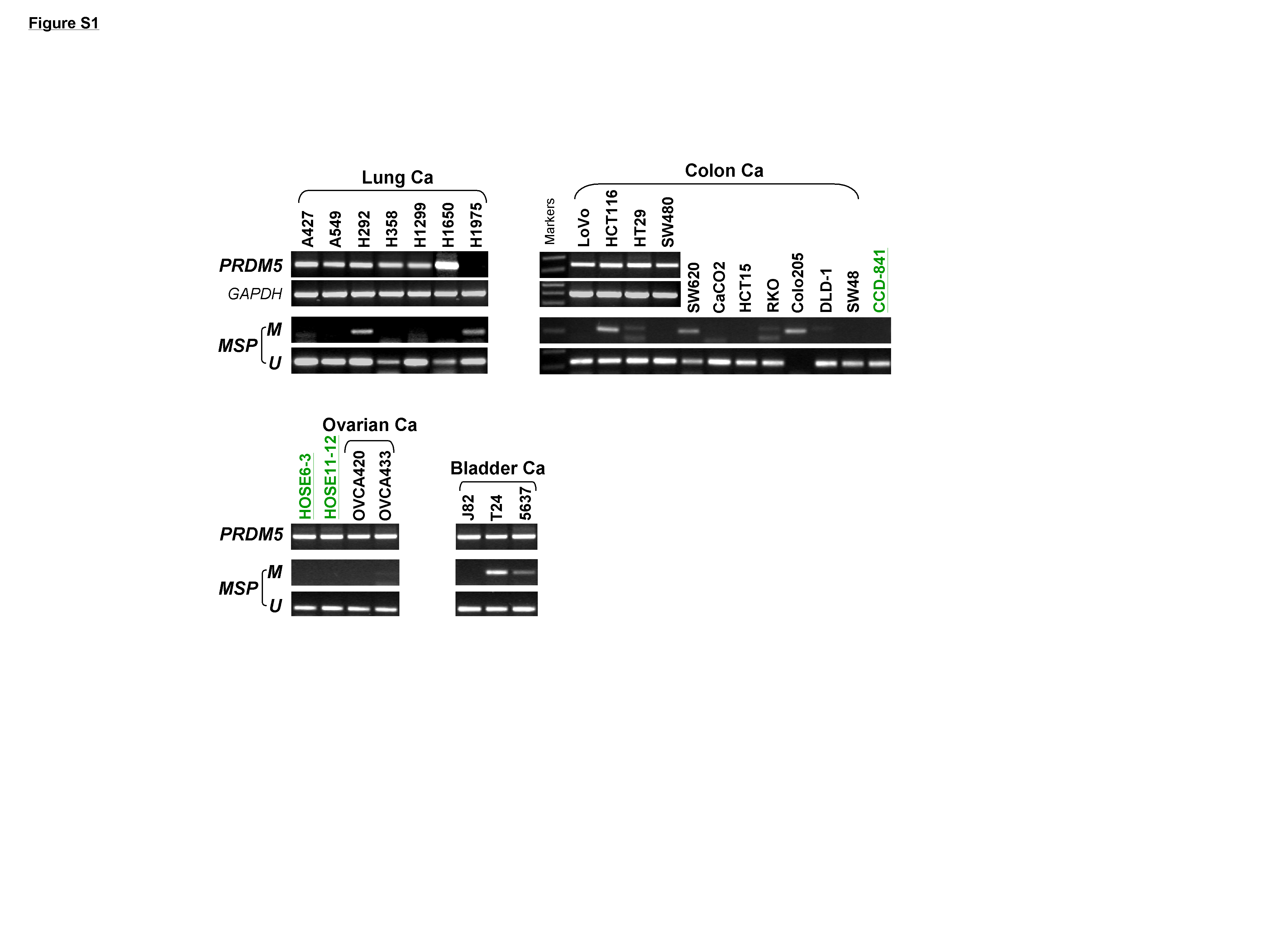

Supplement: Figure S1 — PRDM5 downregulation and methylation is rarely detected in cell lines of lung, colorectal, ovarian and bladder cancer. Ca, carcinoma; M, methylated; U, unmethylated. (TIF) [file pone.0027346.s001.tif]
