# Supplementary material for: The Epigenetic Modifier PRDM5 Functions as a Tumor Suppressor through Modulating WNT/β-Catenin Signaling and Is Frequently Silenced in Multiple Tumors
Source: PLoS One. 2011 Nov 8;6(11):e27346. doi: 10.1371/journal.pone.0027346 (PMC3210799; doi:10.1371/journal.pone.0027346)
Supplement: Table S1 — Real-time PCR of PRDM5 target genes. Modulation of multiple genes by PRDM5 expression in HEK293 and HONE1 cells was evaluated by real-time PCR. Data were obtained from three independent experiments and presented as relative average ratio. The expression of target genes in PRDM5-transfected cells were normalized to those in vector controls, GAPDH was used as internal control. p<0.05 was considered as statistical significance by Student's t-test. (DOC) [file pone.0027346.s002.doc]

Table S1. Real-time PCR of PRDM5 target genes

|  | **HEK293** | | | **HONE1** | | |
| --- | --- | --- | --- | --- | --- | --- |
| **Gene symbol** | **Average ratio  (2^-ddCt)** | **SD** | ***p* value** | **Average ratio  (2^-ddCt)** | **SD** | ***p* value** |
| **Known or candidate oncogenes** | | | | | | |
| **ADAMTS6** | **0.77** | **0.11** | **0.02** | **0.54** | **0.07** | **0.00** |
| **BMI1** | 1.11 | 0.18 | 0.36 | 1.73 | 2.12 | 0.58 |
| **CCND1** | 1.55 | 0.93 | 0.37 | 0.69 | 0.14 | 0.02 |
| **CCND2** | 0.60 | 0.31 | 0.09 | 1.21 | 0.35 | 0.37 |
| **CDK2** | 0.50 | 0.39 | 0.09 | 0.86 | 0.70 | 0.74 |
| **CDK4** | **0.39** | **0.12** | **0.00** | **0.33** | **0.14** | **0.00** |
| **CDK6** | 0.49 | 0.39 | 0.09 | 0.87 | 0.39 | 0.58 |
| **CDK8** | 0.69 | 0.36 | 0.21 | 1.21 | 0.66 | 0.61 |
| **CHD1L** | 0.47 | 0.34 | 0.06 | 0.99 | 0.54 | 0.99 |
| **E2F3** | 0.93 | 0.69 | 0.86 | 0.91 | 0.32 | 0.66 |
| **EZH2** | 0.93 | 0.83 | 0.90 | 1.82 | 1.65 | 0.44 |
| **HDAC9** | 0.71 | 0.26 | 0.12 | 1.05 | 0.37 | 0.84 |
| **ID1** | 0.37 | 0.46 | 0.08 | 0.96 | 0.13 | 0.63 |
| **ID2** | 0.65 | 0.04 | 0.00 | 0.78 | 0.21 | 0.14 |
| **JMJD2C/GASC1** | 0.54 | 0.21 | 0.02 | 0.89 | 0.51 | 0.72 |
| **MDM2** | **0.51** | **0.12** | **0.00** | **0.36** | **0.21** | **0.01** |
| **MYBL1** | 0.52 | 0.30 | 0.05 | 0.77 | 0.24 | 0.17 |
| **MYC** | 0.76 | 0.18 | 0.08 | 1.10 | 0.74 | 0.83 |
| **STAT3** | 0.63 | 0.51 | 0.28 | 0.86 | 0.17 | 0.23 |
| **STAT5B** | 0.39 | 0.11 | 0.00 | 1.35 | 0.63 | 0.39 |
| **TGFB2** | 0.75 | 0.19 | 0.09 | 1.00 | 0.11 | 0.95 |
| **TRIB2** | 0.45 | 0.24 | 0.02 | 0.88 | 0.13 | 0.16 |
| **TWIST1** | **0.45** | **0.28** | **0.03** | **0.64** | **0.05** | **0.00** |
| **WNT4** | **0.35** | **0.14** | **0.00** | **0.57** | **0.11** | **0.00** |
| **WNT10B** | 0.58 | 0.14 | 0.01 | 1.13 | 0.38 | 0.59 |
| **Known or candidate tumor suppressor genes** | | | | | | |
| **BAX** | 0.44 | 0.13 | 0.00 | 0.73 | 0.44 | 0.34 |
| **DKK1** | 1.00 | 0.51 | 1.00 | 0.92 | 0.18 | 0.50 |
| **KISS1** | 1.15 | 0.44 | 0.58 | 0.44 | 0.29 | 0.03 |
| **p27** | 0.56 | 0.25 | 0.04 | 0.90 | 0.22 | 0.46 |
| **PAX6** | 0.71 | 0.09 | 0.01 | 0.83 | 0.54 | 0.62 |
| **PIAS3** | 0.47 | 0.14 | 0.00 | 1.52 | 0.09 | 0.00 |
| **RHOBTB3** | 0.78 | 0.47 | 0.46 | 1.21 | 0.56 | 0.55 |
| **SOCS3** | 0.56 | 0.05 | 0.00 | 0.98 | 0.16 | 0.80 |
| **TP53** | 1.17 | 0.10 | 0.04 | 1.21 | 0.33 | 0.33 |
