# Supplementary material for: The Epigenetic Modifier PRDM5 Functions as a Tumor Suppressor through Modulating WNT/β-Catenin Signaling and Is Frequently Silenced in Multiple Tumors
Source: PLoS One. 2011 Nov 8;6(11):e27346. doi: 10.1371/journal.pone.0027346 (PMC3210799; doi:10.1371/journal.pone.0027346)
Supplement: Table S2 — Primers for target gene screening by real-time PCR analysis. (DOC) [file pone.0027346.s003.doc]

Table S2. Primers for target gene screening by real-time PCR analysis

| **Gene symbol** | **Primers** | **Sequences (5’-3’)** |
| --- | --- | --- |
| ADAMTS6 | ADAMTS6F | TGTGGAAGACGTTGACCTGGA |
|  | ADAMTS6R | AGATACTGCCTGCTGTGGATC |
| BAX | BAXF | CCAGCTCTGAGCAGATCATG |
|  | BAXR | ATGTCAGCTGCCACTCGGAA |
| BMI1 | BMI1-F | GATCACTGAGCTAAATCCCC |
|  | BMI1-R | CAGAAGGATGAGCTGCATAAA |
| CCND1 | CCND1F | TGCTGCGAAGTGGAAACCAT |
|  | CCND1R | GCGGTCCAGGTAGTTCATG |
| CCND2 | CCND2F | TCCGCAGTGCTCCTACTTCA |
|  | CCND2R | GGAGGCCAGGAACATGCAGA |
| CDK2 | CDK2F | AGGTGGAAAAGATCGGAGAG |
|  | CDK2R | GAGCAGAGGCATCCATGAAT |
| CDK4 | CDK4F | CTGTGCCACATCCCGAACTG |
|  | CDK4R | TGGTAGCTGTAGATTCTGGC |
| CDK6 | CDK6F | CGACTTGAAGAACGGAGGCC |
|  | CDK6R | GTGGTCAAGTCTTGATCGAC |
| CDK8 | CDK8F | GACTTTAAAGTGAAGCTGAGC |
|  | CDK8R | CTATTTCTCTACATGCCGACA |
| CHD1L | CHD1LF | ACTGCGGCTTCATACTGAGG |
|  | CHD1LR | CCCAGGATACAGCCATTCTG |
| DKK1 | DKK1F | CTGCATGCGTCACGCTATGT |
|  | DKK1R | AGGTGGTTCTTCTGGAATAC |
| E2F3 | E2F3F | TCTACACCACGCCGCACGGA |
|  | E2F3R | TCTGGACTTCGTAGTGCAGC |
| EZH2 | EZH2F | AGGGACCAGTTTGTTGGCG |
|  | EZH2R | GGGATGACTTGTGTTGGAAAA |
| HDAC9 | HDAC9F | GCACAGTATGATCAGCTCAG |
|  | HDAC9R | GGTGCTGCCGTGTCAAGTTC |
| ID1 | ID1F | TGGAGATTCTCCAGCACGTC |
|  | ID1R | ATGCGATCGTCCGCAGGAAC |
| ID2 | ID2F | ACATCTTGGACCTGCAGATC |
|  | ID2R | AGTGCTTTGCTGTCATTTGAC |
| JMJD2C | JMJD2CF | AAGATAATGACCTTCAGACCC |
|  | JMJD2CR | GAACTCCTTCACAGTCATCG |
| KISS1 | KISS1F | CAGCTACTGCTTTTCCTCTGT |
|  | KISS1R | AGCTGGCTTCCTCTCGGTGCA |
| MDM2 | MDM2F | GCAATACCAACATGTCTGTAC |
|  | MDM2R | CTTGGCACGCCAAACAAATC |
| MYBL1 | MYBL1F | GGATGAGGATGATGACCTTC |
|  | MYBL1R | CTGGCACTGAAAATCAGAGC |
| MYC | MYCF | CTCTCCGTCCTCGGATTCTC |
|  | MYCR | GCCTCCAGCAGAAGGTGATC |
| p27 | p27F | ACTCTGAGGACACGCATTTG |
|  | p27R | TCTGAGGCCAGGCTTCTTG |
| PAX6 | PAX6F | GCCAGAGCCAGCATGCAG |
|  | PAX6R | AGCCAGTCTCGTAATACCTG |
| PIAS3 | PIAS3F | TGCACCCTGATGTCACCATG |
|  | PIAS3R | TGAGGGCAAAGGTAAAGTGC |
| RHOBTB3 | RHOBTB3F | CTGCCCTTGGATTTGAGATC |
|  | RHOBTB3R | CAGCTTGACATTCCCAAACG |
| SOCS3 | SOCS3F | AAGGACGGAGACTTCGATTC |
|  | SOCS3R | GCTGGTACTCGCTCTTGGAG |
| STAT3 | STAT3F | CCAATGGAATCAGCTACAGC |
|  | STAT3R | GCTGATAGAGAACATTCGACTC |
| STAT5B | STAT5BF | ACAAGCTCAGCAGCTCCAAG |
|  | STAT5BR | TGGGTGGCCTTAATGTTCTC |
| TGFB2 | TGFB2F | ACAGCACCAGGGACTTGCTC |
|  | TGFB2R | CGTTGTTCAGGCACTCTGGC |
| TP53 | p53F2 | AAGCAGTCACAGCACATGAC |
|  | p53R2 | TAGTGGATGGTGGTACAGTC |
| TRIB2 | TRIB2F | GCCAGACTGTTCTACCAGA |
|  | TRIB2R | GCAGAATGTAGGCGTCTTC |
| TWIST1 | TWIST1F | TCGACTTCCTCTACCAGGTC |
|  | TWIST1R | CCAGAGTCTCTAGACTGTCC |
| WNT4 | WNT4F | GTCTTCGCCGTCTTCTCAG |
|  | WNT4R | GTACTGGCACTCCTCAATG |
| WNT10B | WNT10BF2 | GGAAGCGGTGAAGAGGAGTG |
|  | WNT10BR2 | GCTCCTCCAGCATGTCGAAG |
| GAPDH | GAPDHF | GATGACCTTGCCCACAGCCT |
|  | GAPDHR | ATCTCTGCCCCCTCTGCTGA |
